# Comparing early life nutritional sources and human milk feeding practices: personalized and dynamic nutrition supports infant gut microbiome development and immune system maturation

**DOI:** 10.1080/19490976.2023.2190305

**Published:** 2023-04-13

**Authors:** Spencer R. Ames, Larisa C. Lotoski, Meghan B. Azad

**Affiliations:** a Manitoba Interdisciplinary Lactation Centre (MILC), Children’s Hospital Research Institute of Manitoba, Winnipeg, Canada; b Department of Immunology, University of Manitoba, Winnipeg, Canada; c Department of Pediatrics and Child Health, University of Manitoba, Winnipeg, Canada

**Keywords:** Immune system development, breastfeeding, human milk, breast milk, infant formula, donor human milk, infant microbiome, weaning reaction, epigenetics, developmental origins of chronic disease

## Abstract

Exclusive breastfeeding is recommended for the first six months of life, but many infants receive pumped milk, formula, donor human milk, or other nutritional sources during this critical period. Substantive evidence shows early nutrition influences development of the microbiome and immune system, affecting lifelong health. However, the underlying mechanisms are unclear and the nuances of human milk feeding are rarely considered. This review synthesizes evidence from human studies and model systems to discuss the impact of different nutritional sources on co-development of the gut microbiome, antigen tolerance, and immunity. We highlight two key mechanisms: epigenetics and the so-called “weaning reaction”. Collectively, this evidence highlights i) the fundamental role of parents’ own milk, fed directly at the breast, as a dynamic and personalized nutrition source that drives developmental programming, and ii) the deficiencies of alternative nutritional sources and priority research areas for improving these alternatives when direct breastfeeding is not possible.

## Plain Language Summary

Before they begin eating solid foods, infants may be fed a variety of nutritional sources such as breast milk (“parent’s own milk”, fed directly at the breast or pumped and fed from a bottle), commercial infant formula, or sterilized “donor human milk” from a certified milk bank. Early nutrition affects the infant’s gut microbiome (bacteria living in the intestinal tract), development of their immune system, and their health throughout life. However, it is unclear how different forms of early nutrition affect these important processes. Parent’s own milk contains compounds that support gut microbes and stimulate development of the infant immune system, changing over time to meet infant needs. For many of these compounds, donor human milk contains a lesser amount, and formula contains even less – or none at all. Some compounds are also affected by pumping and storing parents’ own milk. This review highlights how differences among these nutritional sources influence gene expression and gut development to shape the infant microbiome and immune system. Current evidence shows that parent’s own milk, fed at the breast, offers unique benefits that are not replicated by other forms of early nutrition. This review also outlines how early life nutrition research can help us understand human development and develop new ways to provide the best possible start to life for all infants.

## Introduction

Immune system structures and functions are immature, or differentially adapted, at birth.^1,2^ Compared to adults, neonatal neutrophils, monocytes, and dendritic cells have reduced functional capacity to prevent and clear pathogenic infections.^3–5^ Furthermore, intestinal barriers have increased permeability for up to 6 months, allowing ingested macromolecules, including immunoglobulins (Igs), to translocate across the immature intestinal epithelium.^2,6^ Activated and functional T and B cells are detected around 12 weeks after birth, but at this stage they are more likely to induce tolerance for an antigen rather than clearing it from the infant system.^7–9^ In parallel, the infant microbiome rapidly matures in the first few weeks and months of life. Gut microbiota play a key role in shaping the immune system by protecting the infant from harmful pathogens, facilitating mucosal immune structure stimulation, and inducing antigen tolerance.^10^

During this critical period of early development, infant nutrition influences developmental processes that drive the maturation of the gut microbiome and infant immune system toward the composition and capabilities of a healthy adult.^11–13^ Deciphering how different sources of early nutrition – namely, human milk in various forms, and human milk substitutes of various formulations – influence gut microbiome development and immune system maturation is essential to understanding how to provide the best start to life for all infants.

### Gut microbiome and immune development

Gut microbiome composition varies throughout life and has been implicated in the pathophysiology of many health conditions including inflammatory bowel disease (IBD), colorectal cancer, obesity, diabetes, and neurological disorders.^14,15^ Early life gut colonization is a critical process that has been associated with lifelong gut microbiome composition.^11^ The gut is initially colonized at birth and evolves in terms of diversity and abundance of microbes over time. This process is affected by factors such as birth mode, diet (nutrition and feeding practices), presence of siblings and household pets, antibiotic exposure, and geographical location.^1,10^ Many studies have consistently shown that human milk feeding (i.e. breastfeeding) is the strongest predictor of gut microbiota composition in the first months of life^16–20^ – although very few have distinguished among different forms of human milk feeding.

Gut microbiota confer many helpful and protective immune functions to their host throughout life. Commensal microbes help prevent pathogenic gut colonization and intestinal barrier breaches by competitively excluding pathogens and stimulating epithelial production of antimicrobial peptides.^21^ Furthermore, commensal gut microbes in early life (e.g. *Bifidobacterium infantis, Bifidobacterium bifidum, Lactobacillus rhamnosus)* can enhance gut barrier development by strengthening tight junctions in intestinal epithelial cells.^22,23^ Other common gut microbes, such as *Bacteroides thetaiotaomicron* and *Lactobacillus reuteri*, dampen host inflammatory responses by inhibiting nuclear factor-κB (NF-κB) activation.^24^ This promotes immune system homeostasis throughout life by regulating pro- and anti-inflammatory immune system responses.^25^ Furthermore, gut microbial antigen – immune system interactions in early life are critical for infant antigen tolerance development.^26^ Defects in tolerance induction have been associated with the development of type 1 diabetes, allergy, IBD, and cancer.^26,27^

Certain gut microbes (e.g. some *Bifidobacterium* and *Bacteroides* species) can metabolize dietary oligosaccharides that are non-digestible to the host, including human milk oligosaccharides (HMOs). Microbial products generated from this metabolism (e.g. short-chain fatty acids, acetate, propionate, butyrate, folate, amino acids, vitamins, hydrogen, and carbon dioxide) have nutritional value to the host and can also be used as substrates for enzymes including acetylases, methylases, and glucuronidases.^24,28,29^ These metabolites have also been associated with antigen tolerance regulation, intestinal barrier maturation, and epigenetic modifications within intestinal cells and peripheral tissues – processes that, when disrupted, are associated with the development of pathologies including IBD, obesity, and diabetes.^29,30^

During early life, irregular colonization or antibiotic-induced dysregulation of the microbiome can bias the infant immune system to a hypersensitive or allergic state.^31^ The absence of adequate colonization in early life can lead to an increased likelihood of developing immune-mediated diseases, such as allergy, autoimmune conditions, and type 1 diabetes.^31^ Thus, understanding how early life nutrition influences the gut microbiome is crucial to developing methods for correcting microbiome “dysbiosis” and preventing immune-mediated diseases.

### Forms of early life nutrition

The most common forms of infant nutrition are the birthing parent’s own milk (POM, also known as “mother’s own milk”) and commercial infant formula, typically made from cow’s milk. POM can be fed directly at the breast (DPOM), or expressed and fed from a bottle (EPOM). A less common but increasingly available POM alternative is donor human milk (DHM), which is received, pasteurized, and distributed by a human milk bank.^32^ Informal milk sharing through unregulated networks is another increasingly common practice, but is not discussed in this review.^32^ These nutritional sources vary somewhat in their nutrient profiles and greatly in their dynamism and content of microbial and immunomodulatory factors.^33^

#### Parents’ own milk (POM) – direct or expressed

**Figure 1.**
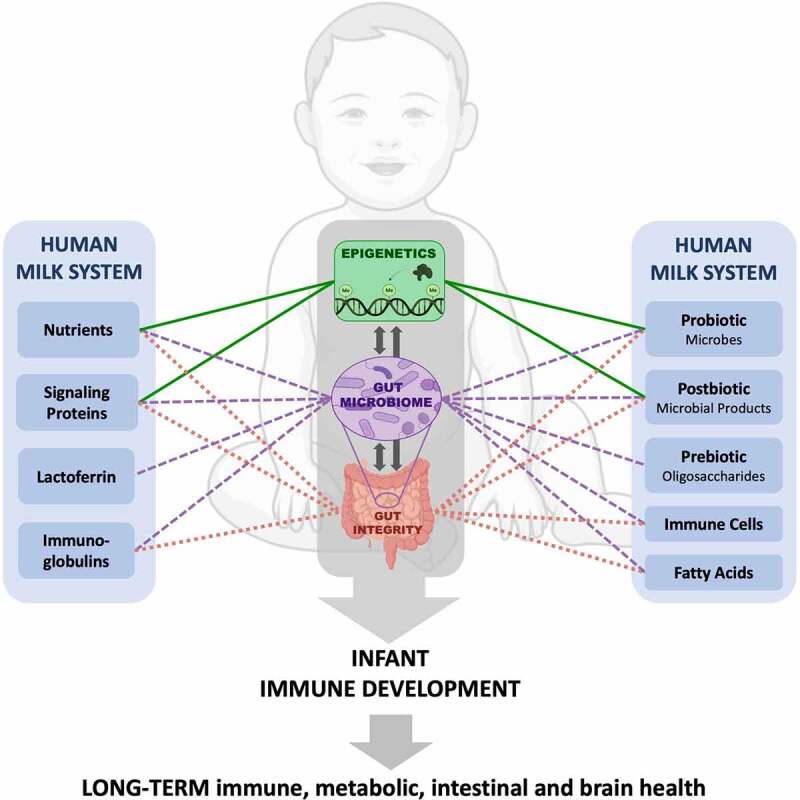
Associations between human milk components and physiological processes that influence infant immune development: epigenetics, microbiome, and gut integrity.

POM is the ‘gold standard’ for infant nutrition. Exclusive POM feeding is associated with a lower incidence of respiratory infections in infancy and childhood, and healthier growth profiles, compared to formula feeding.^34^ A complex and dynamic biological system,^35,36^ POM contains protective and immunomodulatory components, adapts dynamically to infant needs and contains sufficient macro and micronutrients (excluding Vitamin K and D) to fully support healthy infant development for the first six months of life (Table 1, Figure 1).^9,33^ For about five days after delivery, lactating parents produce colostrum, an early form of POM that is low in fat, high in protein, and rich in immunomodulatory components (e.g. bioactive enzymes and immune cells) (Table 1).^33,37^ POM then transitions to a higher fat, lower protein mature milk (>14 days after birth) that generally contains lower levels of immunomodulatory components.^37^ Most immunomodulatory component concentrations within POM continue to gradually decrease until the cessation of POM feeding.^9,33^ These dynamic changes allow the developing infant immune system to gain more “responsibility” and independence in managing and mounting immune responses, setting up healthy lifelong immune function.

**Table 1.** Milk components associated with infant immune system and/or gut microbiome development, and their relative concentrations in different nutritional sources: parents own milk (POM, distinguishing between colostrum and mature milk), frozen expressed POM (EPOM), donor human milk (DHM), and commercial infant formula.

	Parents’ Own Milk (POM)			Donor Human Milk (DHM)^a^	Commercial Infant Formula^b^	Reference
Components	Colostrum (<5 days)	Mature Milk (>14 days)	Expressed & Frozen^c^			
**Immune Cells**						
Macrophages	+++	++ >	Exp, Frz	-	-	38,39,40
Monocytes	+++	++ >	Frz	-	-	39,40
Dendritic Cells	+++	++ >	Frz	?	-	39,40
Neutrophils	+++	++ >	Frz	-	-	39,40
Lymphocytes	+++	++ >	Frz	-	-	39,40
**Immunoglobulins**						
SIgA	+++	++ <	Unaffected	+ ~	-	39,41,42,43
IgG	+++	++ <	Unaffected	+ ~	+ (B)	39,41,42,43,44
IgM	+++	++	Unaffected	+ ~	-	39,41,42,43
**Signaling Proteins and Bioactive Enzymes**						
TGF-ß1	+++	++ >	Unaffected	++ ~	-	39,45,46,47
TGF-ß2	+++	++ >	Unaffected	++ ~	-	39,45,47
IL-10	+++	++ >	Unaffected	+ ~	-	45,48,49
IL-6	+++	++ >	Unaffected	+ ~	-	45,48,50
IL-1ß	+++	++ >	?	+ ~	-	48,50
IGF-1	+++	++ >	?	+ ~	-	39,51
IL-7	+++	++ >	?	?	-	52
TNF-α	+++	++ >	Exp	+ ~	-	38,45,50
GCSF	+++	++ >	?	++ ~	-	48,53
EGF	+++	++ >	Unaffected	++ ~	-	39,45,54,55
Lactoferrin	++++	+++ >	Unaffected	+ ~	+ (B, S)	33,41,42,56
**Microbes**	++	+++ <	Frz	-	- (S)	39,42,57,58
**Prebiotic Oligosaccharides**	+++	++ >	Unaffected	++ ~	- (S)	59–62
**Fatty Acids**						
Short-Chain	+	++ <	Frz	++ ~	+ (B)	60,63–66
Medium-Chain	+	++ <	Frz	++ ~	++ (B, S)	63,64,66,67
Essential Long-Chain (e.g. DHA, ARA)	++	+ >	Frz	++ ~	+ (B, S)	60,63–66
**Postbiotics**^d^	++	+++ <	Frz	++++ ~	+ (B)	57,63,68,69
**MicroRNAs**	++++	+++ >	Unaffected	++ ~	+ (B)	70–73

^a^Difference when frozen at −20°C for 3 months.

^b^Refers to formula made from cow’s milk; the most common type of commercial formula.

^c^Refers to the combination of non-viable microbes and microbial metabolites.

^d^Assuming Holder Pasteurization (62.5°C for 30 minutes).

**Symbols**: (?), unknown; (+ to ++++) relative concentrations from lowest to highest; (-) not detected or not functional; (>) concentration generally decreases over time; (<) concentration generally increases over time; (Exp) affected by expression and/or lack of suckling); (Frz), affected by freezing; (~) concentration varies depending on lactation stage of donated milk; (B) bovine origin, structurally different from human versions; (S) some formulas contain these components as supplemental additives, often in lower concentrations than what is found in human POM.

**Abbreviations**: Ig, immunoglobulin; TGF, transforming growth factor; IL, interleukin; IGF, insulin-like growth factor; TNF; tumor necrosis factor; GCSF, granulocyte colony-stimulating factor; EGF, epidermal growth factor, DHA; docosahexaenoic acid, ARA; arachidonic acid.

POM also contains live microbes that may influence the infant gut microbiome composition.^74^ The composition of POM and its microbiota depend on many dynamic factors, including method of feeding (e.g. directly from the breast/chest, or pumped/expressed and bottled), lactation stage, lactating parent BMI, mode of delivery, infant sex, and geographical location.^75,76^

Interestingly, the level of macrophages and TNF-α within DPOM have been shown to increase when nursing infants are mounting an immune response against an infection, even when the lactating parent is asymptomatic, indicating that DPOM can adapt to the immunological needs of the infant.^38^ The authors hypothesize that this dynamic ability is facilitated by a bi-directional exchange of immune factors that occurs through direct suckling at the breast.^38^ EPOM does not appear to adapt in this manner, presumably because it is collected without direct infant contact and/or due to storage practices that may change its bioactive profile.^38,77^

#### Donor human milk (DHM)

DHM is considered the next best option when POM is unavailable, particularly for vulnerable neonates at high risk of severe morbidity and mortality. The World Health Organization recommends DHM for low- and very-low-birthweight infants, as well as small and sick neonates that cannot receive DPOM.^78^ The eligibility for infants to receive DHM varies regionally and among health care institutions, and the guidelines and policies for the quality, safety, and ethical standards of DHM vary between countries.^79^ There are an estimated 756 milk banks in 66 countries that manage the collection, processing, and distribution of DHM globally.

Although methodologies vary, all certified human milk banks process donated milk to destroy non-spore forming microbes.^41^ The Holder pasteurization process (62.5°C for 30 min) is commonly used to eliminate potential pathogens from donated milk; however, this unfortunately eliminates beneficial milk microbes and immune cells, and lowers the activity of many signaling proteins and bioactive enzymes, such as lactoferrin, lipase and amylase (Table 1). Despite these modifications, pasteurized DHM contains viable forms of many heat-stable immunomodulatory components present in POM, such as Igs and HMOs.^39,59^ Like POM, DHM composition and nutrient content is influenced by donor factors (e.g. lactation stage, diet, age, ethnicity).^33^ Improving DHM processing technology to maximize the preservation of immunomodulatory functions is an active area of research.^80^

#### Infant formula

Commercial infant formula, typically made from cow’s milk, is an option for parents that cannot readily provide POM. While composition varies to some extent between manufacturers, all contain similar nutrient profiles because infant formulas are tightly regulated compared to other food products, although regulations vary among countries.^81,82^ Currently, these regulations do not require the addition of immunomodulatory components (e.g. signaling proteins, bioactive enzymes, immunoglobulins, oligosaccharides, probiotic microbes, postbiotic microbial products)^82^, reflecting the uncertainty regarding the benefits, risks and feasibility of including such additives. Some immunomodulatory components (e.g. oligosaccharides, lactoferrin, and microRNAs) are present or added to some formulas; however, their structures are not identical to the human varieties, and their levels and bioactivity are impacted by the many steps involved in processing cow’s milk to generate infant formula (i.e. pasteurization, homogenization, fractionation, heat treatment, mixing, and emulsification).^56,60,70,83,84^

In addition to its compositional differences from human milk, formula does not have the dynamic ability of POM, so it cannot be optimally matched to the evolving stages of infant development. Reflecting these deficits compared to POM, formula feeding is associated with an increased risk of developing necrotizing enterocolitis (NEC), respiratory infections, asthma, obesity, diabetes, and IBD compared to exclusive POM feeding.^33,60^ Thus, improving infant formula and investigating the short and long-term effects of incorporating human milk components is an important field of infant nutrition research,^60^ alongside supporting, protecting, and promoting POM feeding as the ideal scenario.^85^

### Review aim

This review discusses differences in early life nutritional sources – including POM (DPOM and EPOM), DHM, and commercial formula – and how they impact gut microbiome development and infant immune system maturation. We also comment on the impacts of these nutritional sources and gut microbiota on the weaning reaction and early life epigenetics.

## Compositional differences among different early life nutrition sources that influence microbiome and immune development

**Figure 2.**
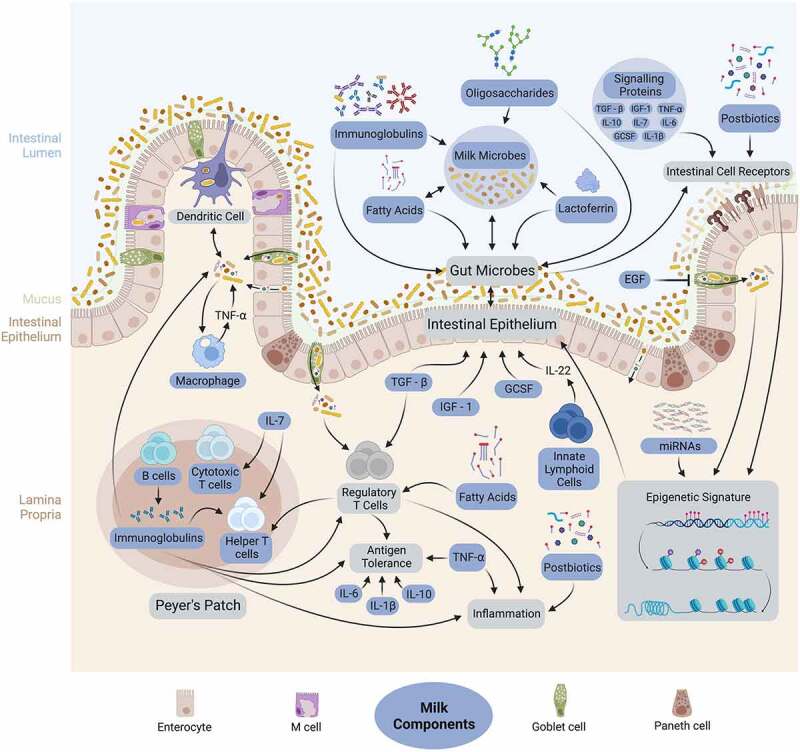
Suggested mechanisms of action and interactions among parents’ own milk (POM) components, infant gut microbiota, and the infant immune system. Adapted from “intestinal immune system (small intestine)”, by Biorender.Com (2023). Retrieved from https://app.biorender.com/biorender-templates.

POM, DHM, and commercial formula all contain the essential macro and micronutrients required for healthy infant development and growth; however, only the POM nutrient profile changes over time as the infant ages. Non-nutrient components including immune cells, immunoglobulins, signaling proteins, and bioactive enzymes are provided through POM (Figures 1, 2), variably present (often at relatively lower levels) in DHM, and mostly absent from formula (Table 1). These differences are important to elucidate because these bioactive components are known to influence infant gut microbiome and immune system development,^57,86^ as discussed below.

### Immune cells

Immune cells (also known as leukocytes) are uniquely present in POM and predominantly consist of dendritic cells, monocytes, macrophages, neutrophils, innate lymphoid cells, and lymphocytes (T cells and B cells) (Table 1).^87^ Their levels follow a predictable pattern after delivery, with concentrations and effector functions of immune cells being highest in colostrum, and gradually decreasing as milk matures.^9,37^ The high buffering capacity of human milk protects these cells from infant saliva-mediated enzymatic digestion and enables them to survive in the infant digestive tract.^87^ Once they reach the infant gut, POM immune cells can infiltrate the permeable neonatal intestinal barrier, allowing them to become activated and motile in the infant.^40^ For example, POM cytotoxic T cells and plasma B cells elicit cytotoxic effects and secrete IgG in the infant, respectively, which may compensate for the decreased activity of immature infant immune cells.^88,89^ Additionally, POM macrophages are phagocytic, secrete cytolytic and inflammatory mediators, and express activation markers (e.g. CD11c) (Figure 2).^90^

Human milk plasma B cells produce immunoglobulins (discussed further below), which in turn, have roles in blocking pathogenic infection, regulating gut inflammation, and promoting antigen tolerance.^91,92^ This POM B cell activity indirectly regulates the development of infant RAR-related orphan receptor gamma expressing regulatory T cells (RORγt+ Tregs) – cells that contribute to colonic immune system control by regulating T helper cell effector functions and subsequent inflammatory responses (Figure 2).^93^ The critical role of RORγt+ Tregs is illustrated in mice deficient for these cells, who display gut microbiome dysbiosis, increased amounts of inflammatory Th17 cells, and increased risk of colitis.^93–95^

Innate lymphoid cells produce an array of effector cytokines associated with intestinal microbiome development and are present in POM.^96,97^ In mice, innate lymphoid cells are major producers of IL-22 - a cytokine associated with gut barrier maintenance and the expression of antimicrobial proteins (e.g *Reg3, S100)* at mucosal surfaces.^96^ IL-22 producing innate lymphoid cells have been associated with preventing commensal gut microbiota dissemination and subsequent systemic inflammation in mouse pups, therefore, it is reasonable to speculate that POM innate lymphoid cells may act similarly within infants (Figure 2).^87^

Thus, immune cells in POM support the development of infant immune system machinery and promote the development of tolerance to microbial species.^87^ Establishing this host-microbial symbiosis in infancy relieves the burden of constant immune system stimulation by gut microbes, and can lower the likelihood of developing NEC or other inflammatory conditions.^57^ Immune cells are inactivated during DHM processing and formula manufacturing.^39^ The lack of functional immune cells in formula likely contributes to the increased likelihood of these conditions as well as gastrointestinal infections among formula-fed infants.^34^

Additionally, both formula and DHM feeding circumvent the bidirectional ‘feedback loop’ enabled by suckling at the breast, where it has been shown that DPOM macrophage concentrations increase during infant infections.^38^ This is relevant to immune development because it suggests that direct feeding allows infants to communicate their immunological needs to their lactating parent.^38^ The same ‘feedback loop’ deficiency applies to EPOM feeding, which likely also suffers from reduced immune cell activity, depending on milk storage conditions.^77^

### Signaling proteins and bioactive enzymes

POM contains various cytokines, chemokines, growth factors and receptors including transforming growth factor-ß1 (TGF-ß1), TGF-ß2, interleukins IL-10, IL-6, IL-1ß, insulin-like growth factor-1 (IGF-1), IL-7, tumor necrosis factor-α (TNF-α), and granulocyte colony-stimulating factor (GCSF) (Table 1).^9,33^ These signaling proteins are associated with both gut microbiome and immune system development, and can act locally in the intestine through binding cell surface receptors, or systemically by permeating the intestinal barrier (Figures 1, 2).^33^

The TGF-ß family is the most abundant family of cytokines in POM.^33^ TGF-ß proteins induce the differentiation of naïve T cells to antigen-specific Tregs that help maintain intestinal homeostasis, induce tolerance, and regulate inflammation.^98^ POM TGF-ß1 and TGF-ß2 have been associated with neonatal gut microbial colonization and composition.^99^ POM TGF-ß2 has been shown to inhibit pro-inflammatory cytokine production in immature intestinal epithelia, decrease gut permeability, and stimulate epithelial repair mechanisms, suggesting they contribute to shaping the intestinal environment and regulating gut microbiota colonization (Figure 2).^99,100^ TGF-ß proteins may also interact with other bioactive components (e.g. microbes, oligosaccharides, bioactive enzymes) in POM to influence gut microbiome composition.

Several interleukins in POM (i.e. IL-10, IL-6, and IL-1ß) have been associated with the development of neonatal oral tolerance to foods, although it remains unclear if this results from the direct cytokine activity on the infant gastrointestinal tract, or if these cytokines are markers of another immunomodulatory mechanism transmitted through POM.^101^ Additionally, GCSF contributes to intestinal epithelial development by increasing intestinal epithelial cell villi, crypt depth, and proliferation in mice pups.^102^ IGF-1 levels in POM have been associated with both the morphological and functional development of the gastrointestinal tract (Figure 2).^51^ IL-7 levels in POM have been associated with infant thymus size and lymphocyte output.^103^

TNF-α is an inflammatory cytokine normally produced by macrophages/monocytes during acute inflammation,^38^ contributing to immune system reactivity, microbiota-induced immune responses, and moderation of gut microbiota composition moderation.^104^ Interestingly, TNF-α levels in DPOM increase when the infant is mounting an immune response to an infection – a dynamic function that is not possible in DHM or formula, and is likely disrupted in EPOM.^38^

Lactoferrin is present at ~5 g/L in colostrum and decreases to ~2 g/L in mature milk.^105^ Lactoferrin is only produced in small amounts by neonates; therefore, POM is the main source of lactoferrin in POM-fed neonates.^106^ This antimicrobial enzyme contributes to selective infant gut colonization, and can prevent pathogenic intestinal bacterial growth by i) chelating iron with a high affinity and ii) altering gram-negative bacterial outer membranes (Figure 2).^107,108^ Lactoferrin administration to mice pups and human infants has been shown to increase *Bifidobacterium* levels in both the gut and feces, indicating lactoferrin has bifidogenic activity.^109^ Low lactoferrin levels in early life may increase infant susceptibility to aberrant gut microbiome development, sepsis, NEC and enteric infections, as well as diabetes and obesity in later life.^106,108^ Infant formula contains low levels (~1.4 g/L) of bovine lactoferrin, which has a similar, but decreased ability to chelate iron.^56,110^ Additional bovine lactoferrin is added to commercial formula in some countries, and is generally recognized as safe by the Food and Drug Administration.^106^

The pasteurization process involved in DHM preparation leads to 40–85% loss in immunologically detectable concentrations of lactoferrin, and IGF-1.^39,41,51^ This suggests lactoferrin, and potentially other signaling proteins and bioactive enzymes present in DHM, are denatured or lose bioactive activity.^39^ The bioactivity of signaling proteins and enzymes in EPOM compared to DPOM has not been widely studied, but likely varies according to each component’s kinetics and sensitivity to different storage conditions.^45^ Bovine signaling proteins and enzymes are largely destroyed by infant formula manufacturing processes (e.g. enzymatic hydrolysis, fermentation, heat treatment, radiation).^83^

### Immunoglobulins

Immunoglobulins (Igs) - including IgA, soluble IgA [SIgA], IgG, IgM, IgE, and IgD – are present in POM, where they resist infant digestive pathways and maintain function in infants (Table 1).^111^ These functions are especially critical during the early neonatal period because Ig-containing and producing host cells are not present at birth; appearing between 10 days to eight weeks of age depending on Ig.^112,113^ Thus, young infants rely on innate immune structures (e.g. physical barriers, innate immune cells) and POM antibodies to avoid infection (reviewed in ref.^9^). Igs transferred in milk can support innate structures by conferring the same function as host-made Igs (i.e. providing ‘passive’ immunity), and by indirectly influencing gut microbiome development, as described below.^92^ The most well-studied POM Igs in terms of microbiome and immune development are IgA and IgG.^92^

SIgA is the most abundant Ig in POM and is capable of binding to antigens present on toxins, viruses, and both commensal and pathogenic microbes.^92,114^ SIgA-microbe interactions contribute to ‘beneficial’ early life microbe (e.g. bifidobacteria, lactobacilli) colonization and maintenance within the infant gut.^114,115^ SIgA-microbe interactions are also associated with the prevention of both commensal and pathogenic gut microbe translocation across the mucosal epithelium,^92^ as demonstrated in a mouse model where increased penetration of commensal microbes into mesenteric lymph nodes was observed in pups fed POM lacking IgA, when compared to pups fed POM with IgA.^116^ Treg activity has also been associated with the level of IgA-microbe coating, suggesting IgA-microbe interactions may have a role in modulating how microbes are sensed by the immune system.^114,117^

Furthermore, POM IgG and IgA have been shown to dampen mucosal T helper cell responses in mice, limiting adaptive immune responses to commensal antigens.^118^ Verhasselt et al.^27^ have also proposed that human milk IgG-antigen immune complexes can transfer across intestinal barriers and mediate inflammatory responses by promoting the formation of infant Tregs. POM Ig microbiota exclusion and immune system regulation promotes intestinal homeostasis and helps prevent excessive immune stimulation as the infant gut is colonized by microbes.^92^

DHM contains a lower concentration of functional Igs when compared to POM, attributable to DHM pasteurization and freeze-thaw processes.^39,111^ EPOM Igs may also be affected by freezing, although this has not been widely studied. The lack of immunoglobulins within formula may contribute to the increased levels of gut inflammation observed in formula-fed infants.^60,119^ Additionally, compared to controls, mouse pups that did not receive SIgA in POM had different gut microbiota compositions persisting into adulthood, and higher expression of genes associated with IBD and inflammatory disease, suggesting that lower levels of functional Igs in DHM and formula may have implications for infant development and lifelong health.^92,120^ Understanding the composition and impact of POM Igs, both alone and in conjunction with other POM components, on early life immune system development is an important future research direction.

### Microbes and probiotics

POM contains microbes, including several taxa that populate the infant gut in early life (e.g. *Bifidobacterium* and *Streptococcus*) (Table 1).^76,121^ The diversity and amount of POM microbes change over time^58^ and some taxa are shared between POM and infant stool,^76^ suggesting that milk microbiota may contribute to infant gut colonization (Figure 2).^16–18^ However, the sources and functions of milk microbiota are not well understood, in part because it is methodologically challenging to study milk microbes due to their relatively low abundance. It is also important to note that DNA sequencing methods are not able to differentiate between viable and non-viable bacteria; therefore, alternate methods are required to investigate live microbiota in POM and better understand their functional roles.^122^

Milk from lactating parents who feed EPOM has a different microbial profile than those who do not, suggesting the method of POM feeding (directly at the breast vs. pumped and bottled) is a key factor influencing POM microbiota composition, presumably due to the contribution of exogenous bacteria (e.g. from the infant oral cavity and/or pumping equipment).^123,124^ Investigating the impact of different breast pump cleaning practices and milk storage conditions will help elucidate the relevance of these processes to immune system development.^74,122^

DHM pasteurization and formula processing destroy 99% of non-spore forming microbes.^39,57^ Despite this, DHM feeding is associated with preterm infant gut microbiota composition,^125^ indicating that other factors present in DHM – such as HMOs and Igs – contribute to infant gut microbiota composition. Research is underway to investigate if incubating pasteurized DHM with a sample of POM can amplify POM microbes in DHM, although the clinical feasibility and safety of this method has yet to be examined. Potentially, this method could assist parents struggling to express adequate quantities of milk by introducing their infants to a beneficial cocktail of POM microbes.^126^ Collecting multiple POM samples and incubating in DHM over time could also allow for changes in POM microbiota over time to be reflected in DHM.^127^

Commercial probiotic supplements can be used to add microbes to DHM and formula. *Bifidobacterium* and *Lactobacillus* are commonly included in early life probiotic supplements and are generally recognized as safe by the Food and Drug Administration.^57,128^ Some lactobacilli and bifidobacterial probiotic strains are capable of fermenting HMOs into simpler sugars that can be utilized as an energy source by the host.^129,130^ Additionally, 6–12 month old infants receiving formula supplemented with *Lactobacillus fermentum* (and galactooligosaccharide) have a decreased incidence of gastrointestinal and upper respiratory infections when compared to age-matched control infants (receiving formula supplemented with galactooligosaccharides only).^131^ *Bifidobacterium lactis* formula supplementation has also been associated with enrichment of beneficial early-life bacteria (*Bifidobacterium* and *Lactobacillus*) in low birth weight infants.^132^ Accordingly, probiotics have been proposed as a strategy to improve gut microbiota colonization in low-birth weight infants exposed to antibiotics early in life;^133^ however, there are concerns that large doses of probiotics administered to immunologically immature infants could overwhelm the immune system and lead to infection and sepsis.^134,135^

The optimal composition and safe dosage of probiotic microbes for infants at different stages of development is an important area of ongoing research.^136^ Notably, human milk could be an important source of inspiration for this research because at least some members of the human milk microbiome appear to have co-evolved with our species to survive digestion and seed the infant gut.^76^

### Oligosaccharides

Prebiotics are compounds that are fermented by beneficial microbiota and stimulate their growth. Human milk oligosaccharides (HMOs), the third most abundant component of human milk, serve as prebiotics in the infant gut where they are the preferred substrate for bifidobacteria and other select taxa.^137^ HMOs also act as soluble receptor decoys that bind to pathogenic bacteria and prevent them from infecting the host, and can act locally on mucosa-associated lymphoid tissue to inhibit the expression of inflammatory genes (Figures 1, 2).^137^ More than 200 HMOs have been identified in POM; their concentrations vary during lactation with the majority being most abundant in colostrum (average 9–22 g/L) and gradually decreasing (6–15 g/L in mature milk) until weaning (Table 1).^138^ Two exceptions are 3-fucosyllactose and 3’ sialyllactose, which increase in concentration over the course of lactation.^33,138^ Like other POM components, HMO concentration changes likely occur to match changing infant needs over time. HMO concentration is also influenced by country of origin, genetics, lactating parent health and environmental factors.^138,139^ Unlike many other POM components, HMO concentrations and patterns are not affected by pasteurization or freezing; thus, HMOs are largely preserved in EPOM and DHM.^59^

Recognizing the importance of HMOs to the infant microbiome and health, formula manufacturers are increasingly incorporating prebiotic oligosaccharides into their products. Initially these were limited to fructooligosaccharides (FOS) and galactooligosaccharides (GOS), which are oligomers derived from lactose and inulin, respectively.^140^ In randomized clinical trials of formula-fed infants comparing GOS and/or FOS-enriched formulas to control formulas without these additives, GOS has been shown to enrich *Bifidobacterium* in infant stool^84^ and GOS/FOS reduced the rate of infections in the first six months of life.^140^ Bovine oligosaccharides have also been added to infant formulas, eliciting higher counts of infant stool *Bifidobacterium* and *Lactobacillus* compared with control formula.^141^ More recently, some formulas have begun to incorporate HMOs including synthetic 2’-fucosyllactose and lacto-N-neotetraose, with initial studies focused primarily on establishing the safety of these additives.^142^ More research is required to understand the clinical benefits of adding these ingredients to infant formula, and to explore the value of other combinations of different oligosaccharides. It is important to note that GOS and FOS are structurally and functionally very distinct from HMOs naturally found in human milk,^33,137^ and that even HMO-containing formulas have vastly different HMO compositions compared to human milk (i.e. just one or a few HMOs at relatively low concentrations in formula, compared to the dozens of different and highly abundant HMOs in human milk).^137^

### Fatty acids

Human milk contains fat globules (also known as mature lipid droplets) predominantly composed of fatty acid (FA) bound triacylglycerols.^143^ Mature human milk FA content consists of<1% short-chain FAs, ~12% medium-chain FAs, ~82% long-chain FAs, and~1% “essential” polyunsaturated long-chain FAs. Essential FAs cannot be biologically synthesized by humans and must be acquired from an individual’s diet.^60,143^ Human milk FAs serve as a major energy source for the infant and have essential biological functions contributing to neurological and immune system development, as well as gut microbiota colonization in early life (Figure 2).^144,145^

FAs are antimicrobial – their amphiphilic properties allow them to disrupt or remodel microbial cell membranes by acting as a detergent and solubilizing cell membrane materials, or by inserting into a microbial cell membrane.^143,146^ These mechanisms lead to the inhibition of microbial growth, observed *in vitro* when microbial taxa such as *Lactobacillus*, *Bifidobacterium*, *Escherichia*, and *Clostridium* are cultured with medium-chain or long-chain polyunsaturated FAs.^145,147,148^ Despite uncertain nuances of FA-microbe interactions, including structure-dependent antimicrobial activity differences and potentially positive effects of absorbed FAs on microbial activities,^145^ this evidence suggests that POM-derived long and medium-chain FAs may contribute to the regulation of microbial growth in the gut (Figure 2).

POM FAs have been associated with infant immune function. Laitinen et al.^149^ show that POM received by infants with atopic eczema contained a lower amount of polyunsaturated long-chain FAs compared to control infants. Furthermore, essential polyunsaturated long-chain FAs present in POM, including arachidonic acid and docosahexaenoic acid, induce anti-inflammatory cytokine expression and Treg activation.^150^ These FAs are also important substrates for the synthesis of eicosanoids – signaling molecules with critical roles in immune system processes including platelet aggregation and cell proliferation.^144^ In addition short-chain FAs in POM can serve as energy sources for epithelial cells and stimulate lamina propria Treg proliferation and anti-inflammatory cytokine expression.^151^

Human milk FA profiles change with lactation stage – the concentrations of short and medium-chain FAs increase over time, and the concentrations of essential polyunsaturated long-chain FAs (i.e. docosahexaenoic acid and arachidonic acid) decrease over time.^60,63,143^ The human milk FA profile is also influenced by geographic location, maternal diet and maternal BMI.^63^ The association between POM FAs and immune system activity indicate FA profile changes may occur in a manner suited to the infant’s evolving gut microbiome, immune system, and dietary source.^63^

Human milk FA profiles are not impacted by Holder pasteurization^64^ and appear to remain stable when human milk is frozen at −60°C.^152^ In contrast, freezing human milk at −20°C leads to a decrease in fat content in as few as two days^65^, which has implications for EPOM and DHM as parents and milk banks and often freeze human milk at −20°C.^67^ FA profiles are further impacted by lactation stage – a particularly important consideration for DHM because human milk donors are often term-delivering parents donating mature milk.^111^ In samples collected from an Iowa milk bank, docosahexaenoic acid levels were 53% lower in DHM, compared to colostrum, indicating there may be an increased risk of essential FA deficiency in infants receiving DHM.^66^ To address this concern, it has been suggested that human milk banks could supplement DHM with docosahexaenoic acid, or encourage human milk donors to supplement their diet with docosahexaenoic acid.^66,148^

Most infant formula is derived from cow’s milk and supplemented with vegetable fat blends (e.g. palm, coconut, sunflower, and/or soy oil), as well as manufactured docosahexaenoic acid and arachidonic acid, in an attempt to create a FA profile that closely resembles human milk.^60,153^ However, most vegetable FAs are attached to triglycerides at different structural positions (sn-1 and sn-3) compared to human milk FAs (sn-2).^154^ One study has shown that infants receiving synthetic sn-2 positioned palmitic acid for six weeks had an increased abundance of fecal *Lactobacillus and Bifidobacterium* when compared to infants receiving a control formula.^155^ Optimizing the FA profile of human milk substitutes is an active area of research.

### Postbiotics

Postbiotics refer to non-viable microbes (intact or broken) and/or microbial metabolites that confer a health benefit on the host. This includes cell wall fragments, microbial cell fractions, short-chain fatty acids, enzymes, vitamins, and other bacterial metabolites.^68,156^ Postbiotics have some similar functions to probiotics; for example, they can adhere to gut mucosa, bind to cell receptors, transduce receptor signals, and exclude pathogens from colonizing the gut microbiome. However, they do not actively metabolize prebiotic substrates.^157^ In gnotobiotic mice, *Bifidobacterium breve* postbiotics have been shown to suppress pro-inflammatory cytokine production in the spleen, although live strains had greater impact on the regulation of intestinal metabolism.^158^ Mice receiving infant formula supplemented with postbiotics derived from *Bifidobacterium breve* and *Streptococcus thermophilus* show prolonged dendritic cell survival and maturation, improved epithelial barrier function, and increased anti-inflammatory cytokine (i.e. IL-10) production (Figure 2).^68^ Additionally, metabolic products released by *Lactobacillus paracasei* fermentation, when added to formula, have been shown to inhibit immune cell inflammation and can protect against colitis in mice.^68,159^ Further research is needed to determine if similar effects are seen in humans, but it has been suggested that postbiotics could serve as a safer option to probiotics for particularly vulnerable infants at risk for infection or NEC.^68,156^

Postbiotics can be present in any solution containing microbes, including all forms of human milk (Table 1). Presumably, differences in EPOM and DPOM microbiota composition (described above) would lead to differences in postbiotic composition – although to our knowledge, this has not been studied. The DHM pasteurization process kills viable microbes, suggesting the level of postbiotics in DHM may be higher compared to POM. DHM postbiotic activity may contribute to the maintained protection and stabilization of infants at risk of or suffering from NEC. At the time of this review, however, little has been reported on DHM postbiotics.^160^ Finally, while raw cow’s milk contains postbiotics, formula processing techniques result in protein proteolysis and denaturation, as well as epitope destruction, which likely impact postbiotic presence and functionality.^39^

Further research is required to understand the function of postbiotic compounds and confirm the safety and utility of postbiotic supplementation. This includes investigating their influence on gut microbiome development and immune system maturation.

## Critical processes in early life immune system development affected by early nutrition and the microbiome

Epigenetic modifications and the so-called “weaning reaction” are two critical processes that are affected by early nutrition and gut microbiota, and contribute to immune development. In this section, we describe these key processes and discuss how they may be influenced by different nutritional sources during infancy.

### The weaning reaction

**Figure 3.**
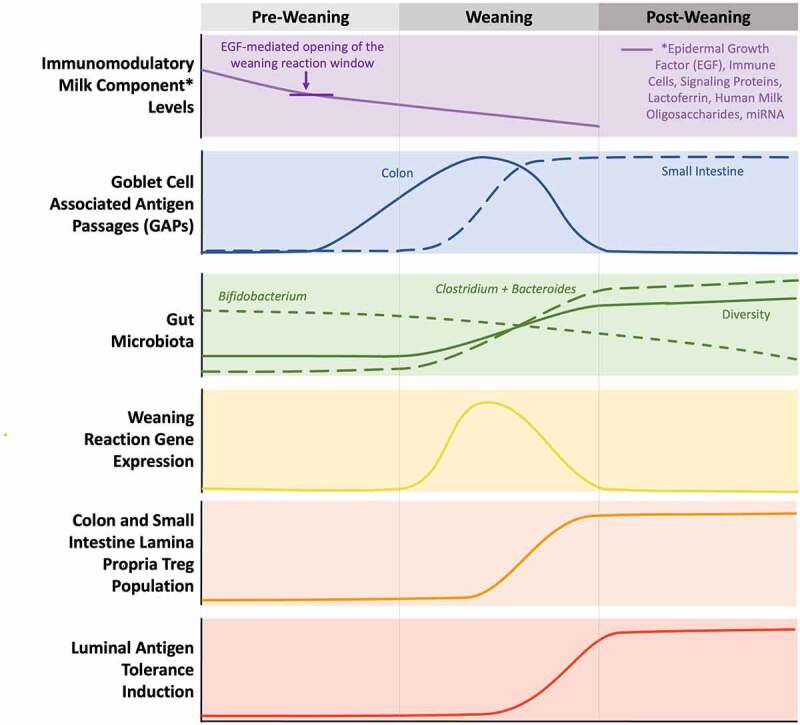
Approximate changes in milk composition and other factors associated with the weaning reaction over time, in mice. Adapted from Hornef and Torow (2019).^1^

Weaning refers to the dietary shift from POM to other foods. Mouse studies show that, during a specific time window, weaning pups undergo vigorous gut microbiota-induced immune stimulation leading to changes in gene expression and microbiome composition in the intestinal tract (Figure 3).^17,161^ Al Nabhani et al.^161^ have shown that this weaning reaction contributes to RORγt+ Treg generation and the prevention of “pathological imprinting” (susceptibility to inflammatory pathologies later in life, including allergy, autoimmunity, IBD, and chronic inflammation). Although mostly studied in mice thus far, the weaning reaction may have a critical role in human immune system development.^26,161,162^

#### Overview of the weaning reaction

Weaning is accompanied by the withdrawal of POM components from the infant system and changes to gut microbiome composition induced by new nutritional sources (Figure 3). During this transition, the loss of POM components appears to induce the formation of goblet cell associated passages (GAPs) in the colon and small intestine.^26,162^ Although other routes of translocation across the luminal epithelium exist, GAPs are the main pathway for delivering luminal substances to the colonic and small intestine lamina propria (the connective tissue that underlies the gut mucosal epithelium).^162^ These luminal substances, including microbial and dietary antigens, stimulate lamina propria immune cells (e.g. mononuclear phagocytes) and facilitate the generation and maintenance of RORγt+ Tregs,^26,162^ which further regulate effector T cells to promote antigen tolerance (Figure 2).^93^ This sequence of developmental processes has lifelong implications, as early-life insufficiency of RORγt+ Tregs is associated with an increased susceptibility to colitis, allergic inflammation, and cancer across the lifecourse.^161^

The weaning reaction also induces widespread changes in gene expression.^161^ Genes induced following the recognition of luminal antigens by innate immune receptors in the gut epithelium include many associated with anti-microbial immunity, such as Reg3 defensins, CC and CXC chemokines, and cytokine receptors. Additionally, the weaning reaction stimulates TNF-α and IFN-γ expression in T cells (Figure 3).

Throughout life, GAPs facilitate the creation and maintenance of lamina propria Tregs by “sampling” luminal antigens^162^, but this process is inhibited periodically when antigen exposure may be harmful to the host. For example, it has been shown in mice that GAP inhibition occurs as a defense mechanism to limit inflammation when the host is infected with *Salmonella*.^163^ Similarly, POM-induced inhibition of GAP formation in early life appears to be an important mechanism for preventing excessive immune system stimulation in young offspring who may be unable to efficiently clear or respond to luminal antigen exposures.^164^

EGF and other EGFR ligands in POM have been proposed as the master regulators of GAP inhibition prior to weaning (Figure 2). In POM-fed mice, who typically wean around 3 weeks of age, POM-derived EGF is thought to inhibit GAP formation in the first 10 days by binding to goblet cell EGFRs (Figure 3).^26,165^ This is supported by i) evidence showing limited presence of luminal antigens in the normal mouse gut during this period,^26^ ii) a cross-fostering study showing that “early milk” blocked the weaning reaction in mouse pups while mature milk did not,^161^ and iii) experiments showing that the weaning reaction was blocked by EGF exposure and induced by EGF receptor inhibition.^161^ Additionally, EGF supplementation has been shown to increase intestinal barrier strength and reduce NEC severity in preterm rats.^166^

Further evidence of the time-sensitive weaning reaction comes from experiments manipulating the timing of microbial exposures in germ-free mice.^161^ Germ-free mouse pups introduced early to microbes that normally expand during weaning (e.g. *Clostridia, Bacteroidia*) are pathologically imprinted (i.e. have an increased susceptibility to inflammatory pathologies) when compared to pups that are exposed to the same microbes during the developmentally appropriate critical time window. Furthermore, germ-free pups introduced to the same microbes after the completion of weaning do not express TNF-α and IFN-γ - two markers of the weaning reaction. Thus, the introduction of microbes before or after the window of opportunity appears problematic for host development. Aside from the timing of exposure, the type of microbial stimulation also appears to be important, as gram-positive anaerobic microbes were necessary to induce the weaning reaction in these germ-free mouse experiments. Together, this evidence indicates that important microbiome-mediated developmental imprinting occurs during a clearly-defined time window that “opens” in response to the withdrawal of EGF in POM (Figure 3), and “closes” after weaning is complete, though the mechanisms of this closure remain to be determined.^26,161^

The weaning reaction has not been clearly defined in humans, but similar to mice, EGF is present at high concentrations in human colostrum (25–40 ng/mL) and gradually decreases as POM matures (5–12 ng/mL in mature milk).^54^ It has been hypothesized that when POM EGF levels drop below the threshold required for GAP inhibition, GAPs form and allow vigorous microbial dissemination into both the small intestine and colonic lamina propria (Figure 3), triggering a critical shift in gene expression and cascade of immune system maturation processes required for optimal development and health.^161^ Notably, this hypothesis suggests that the “weaning reaction” is stimulated by natural changes in POM composition, and could be triggered by the premature withdrawal of POM components, but does not necessarily require complete cessation of POM consumption.

#### Nutritional impacts on the weaning reaction

While EPOM and DHM preparation processes do not have a significant impact on EGF concentrations in milk,^39,45^ they might impact the weaning reaction by disrupting the “chronobiology” of POM feeding.^164^ Human milk donors are most often term-delivering parents donating mature milk,^111^ with relatively lower EGF levels than would be present in POM produced for premature newborns at early stages of development, which may not effectively inhibit GAP formation. Indeed, this has been demonstrated in mice where “early milk” blocked the weaning reaction in recipient pups, whereas mature milk did not.^161^ This supports the proposal by Knoop et al.^164^ that due to temporal changes in POM composition, DHM from donors closer to parturition may provide superior protection compared to DHM expressed later in lactation.

Formula contains little or no EGF.^55^ This is reflected in infant stool, where EGF concentrations are lower over the first 60 days of life among exclusively formula-fed vs. exclusively human milk-fed infants.^164^ While the weaning reaction was not characterized in this human study, it has been shown that mice with low EGF exposure prior to weaning (mimicking the exposure of formula-fed children) have accelerated colon GAP formation, increased bacterial translocation, and an elevated risk of enteric pathogen sepsis.^164^ This suggests the lack of EGF in formula may have implications for health in humans. Indeed, brief periods of formula feeding have been associated with an increased risk of NEC, when compared to infants receiving exclusive POM.^167^ These brief periods without POM EGF may induce GAP formation, luminal antigen exposure among colon and small intestine lamina propria immune cells, and inflammatory responses, potentially explaining the increased risk of NEC associated with brief periods of neonatal formula feeding.

Finally, considering that gut microbes are key mediators of the weaning reaction,^161^ it is also possible that different early life nutritional sources could indirectly influence the weaning reaction through their strong and differential impacts on the gut microbiome in early life. Understanding the strain-specificity of the weaning reaction could help elucidate the role of the gut microbiota in immune system development.

### Early life epigenetic modifications

Early life epigenetic processes regulate gene expression and impact lifelong health, serving as a primary mechanism for developmental programming. The term epigenetics means “above” or “on top of” genetics and refers to the systems that control gene expression without transforming genomic sequences. Epigenetics control gene expression through i) covalent modifications to DNA and histones (e.g. methylation, acetylation and phosphorylation) – known as epigenetic imprinting^168,169^ and ii) microRNA (miRNA) interference, where miRNAs bind to mRNA and disrupt the translation process or signal mRNA degradation. Epigenetic imprinting occurs to the greatest degree from the time of conception to the infant’s second birthday (the first 1000 days of life),^169^ and has been associated with both early life nutrition and the development of conditions such as asthma, allergy, obesity and diabetes later in life.^29^ Gut microbiota influence epigenetic processes that mediate inflammation and intestinal barrier integrity in early life.^169^ Thus, the interplay between infant nutrition, the gut microbiome, and epigenetic modifications is an important relationship to consider when investigating immune system development.

#### Early life nutrition and epigenetics

Nutritional components and by-products (e.g. folate, methionine, choline, vitamin B-12) can modify epigenetic reactions, and the diet is an important source of epigenetically-active miRNAs. Therefore, the variable nutrient profiles and miRNA content of different early life nutritional sources are key determinants of epigenetic programming during infancy.^29,71,168^ Indeed, early life nutritional exposures have been associated with epigenetic modification of genes vital for immune system development and growth, such as *FOXP3* (Treg development), *FTO* (obesity), *INS* (diabetes), *IGF1* (growth).^29,71,169^ In pigs, formula feeding has been associated with decreased amounts of methylation, and increased amounts of hyperacetylation at various histone protein sites responsible for inflammatory gene (IL-8, toll-like receptor (TLR) 4) expression, when compared to POM feeding.^170^ In human intestinal cells, human milk was shown to inhibit the activation pathway of NF-κB.^71,171^ Furthermore, the expression of NF-κB and Foxp3 is down and up-regulated, respectively, by human milk miRNAs.^29,172^ These transcription factors target thousands of other genes, including many that regulate Treg proliferation and immune system development.^173^ Additionally, new evidence shows that intake of POM by very low birth weight infants is associated with DNA methylation patterns at 5.5 years of age.^174^ The epigenetic signatures of other infant feeding practices among full term infants has yet to be established but may provide a deeper understanding of how POM shapes immune system development.

miRNA content and composition vary among human milk sources and substitutes. POM contains over 1400 miRNAs originating from the lactating gland; with concentrations decreasing over the course of lactation (Table 1).^71^ The composition of miRNAs in POM depends on the lactating parent’s diet, gut microbiota, genetics and time postpartum,^71,175^ presumably in response to infant feeding patterns and needs.^176^ These POM miRNAs have been associated with the expression of over 4000 DNA CpG sites – including sites in genes involved in development and function of the immune system (Figure 2). For example, MiR-223 is a common POM miRNA that regulates transcription factors that influence the proliferation of T cells and granulocytes.^71^ Similarly, MiR-155 is also found in POM and regulates Treg development indirectly by regulating Foxp3 transcription factor expression.^172^ Determining additional targets and functions of POM miRNAs is an important direction for future research.

miRNA differences between EPOM and DPOM have not been studied. miRNAs are present in DHM and are resistant to degradation characteristic of pasteurization and milk bank storage.^71^ miRNAs have been detected in cow milk-based formula, but at very low concentrations relative to human POM.^70,177^

#### Early life gut microbiota and epigenetics

Early nutritional exposures can also influence epigenetic modifications through their impact on the gut microbiome.^169^ Two major mechanisms have been suggested to explain how microbiota influence epigenome variation.^178^

(I) Gut microbes can impact host epigenetic modification pathways by interacting directly with components that mediate epigenetic mechanisms.^169,179^ For example, commensal microbiota are recognized by pattern-recognition receptors (PRRs) (e.g. Toll-Like Receptors [TLRs]) on intestinal epithelial cells, triggering the expression of genes with epigenetic activity, such as *Pigp, Hdac9, H3Kac, H4Kac*, and *Tollip* (reviewed in ref.^180^)(Figure 2). Additionally, methylation of the TLR4 gene is lower in germ-free vs. conventionally housed mice,^181^ indicating a role for gut microbiota in mediating this epigenetic modification and the downstream expression of TLR4. These findings highlight a potential mechanism of microbiota-epigenetic-induced tolerance in early life, because TLRs are involved in sensing and stimulating an immune response to environmental stimuli.^181^ Further investigation is required to understand other pattern recognition receptor signal transduction pathways induced by different microbes and elucidate their downstream epigenetic effects. Further research is also required to investigate epigenetic modifications resulting from the incorporation of foreign, microbial genetic material into the host.^178^

(II) Microbial metabolites can alter the availability and function of epigenetic machinery (reviewed in ref.^182^) (Figure 2). For example, butyrate produced by microbes (e.g. select *Lactobacillus*, *Bifidobacterium*, and *Clostridium* species) can act as a histone deacetylase (HDAC) inhibitor.^183^ Butyrate HDAC inhibition can suppress pro-inflammatory NF-κB activation and decrease IFN-γ production.^184^ Lactobacilli and bifidobacteria also produce folate, a cofactor necessary for the transfer of carbons in DNA methylation processes.^183^ Folate and butyrate are also present in POM and DHM (Figure 1).^33^ Without these microbial metabolites, infants can be predisposed to intestinal and systemic inflammation,^178^ which are hallmarks of conditions such as NEC, asthma and allergies.^179^ Early life nutritional sources that promote lactobacilli and bifidobacteria colonization of the infant gut, such as POM and DHM, can regulate inflammation levels in this way.^168,169,179^

Alongside this evidence that gut microbes affect host epigenetics, there is also evidence that epigenetic regulation of epithelial barrier development and intestinal metabolite processing can affect gut colonization. This indicates the presence of a host-microbe cross-talk mechanism in which microbiota influence host epigenetic signatures, and host epigenetics influence microbiota composition – both of which are clearly impacted by early life nutrition and feeding practices, as described above (Figure 1).^178^ More research is needed to gain a deeper understanding of how early life nutrition affects host-microbe cross-talk and it’s resulting impact on host epigenetics and immune development.

## Conclusion

The collective evidence in this review suggests early life POM feeding optimally regulates the colonization and composition of the infant gut microbiome, with both short and long-term health implications. POM components also mediate the *interplay* between the infant gut microbiome and immune system stimulation – a process critical for tolerance induction, healthy immune system development, and prevention of pathological imprinting. Many POM components – from nutrients and enzymes to cells and microbes – have been directly or indirectly linked to these key developmental processes. Temporal changes in POM allow the maturing infant immune system to gradually gain more “responsibility and independence” over time. When fed directly at the breast, POM appears to adapt to the infant’s immunological needs through bi-directional parent-infant signaling. Fully characterizing the extent and significance of POM composition and functionality as a biological system^35^, including its dynamism and adaptability, will help us further understand the fundamental influence of nutrition on additional processes that were beyond the scope of this review, including metabolism and neurodevelopment, as well as overall human development.

This review also highlights the limitations of DPOM substitutes from the perspectives of gut microbiome and immune development. Commercial formula lacks most of the immunomodulatory components found in POM, and is not a dynamic nor personalized form of nutrition. While DHM retains some of these factors, processing steps alter their bioactivity and current DHM provision strategies are not personalized or dynamic. Finally, EPOM feeding is the closest alternative to DPOM and offers many benefits, but affects milk microbiota composition and interferes with the natural dynamism and adaptability of DPOM feeding. Throughout the review, we have identified priority research areas (summarized in Box 1) with the goal of improving all forms of infant feeding to support lifelong immune health.

**Table:** **Box 1.** Priority areas for future research on early life nutrition, gut microbiota and immune development.

Characterizing the full extent of POM components and applying a systems biology approach to determine their independent and collective functions in the infant How do these functions change over lactation? How do these functions contribute to immune system development? How do different POM components interact with each other? Priority components: miRNAs, HMOs, SIgA, EGF
Understanding the viability and function of immune cells and microbes in POM Are they viable? Do they survive digestion? Do they act locally in the gut and/or systemically? What are their functional roles in the infant? Do these roles change over time as the infant gut matures?
Continuing research on the short and long-term effects of adding different POM components to infant formula. Which POM components and combinations are most important to include? How do these components influence microbiome development? How do these components influence immune system development? What composition and dose are safe and efficacious for infant health and development? Is it possible and beneficial to mimic the dynamism and chronobiology of POM?
Improving our understanding of how POM adapts to infant needs, and how direct POM feeding facilitates this adaptability. What POM components can adapt according to infant needs? What infant needs stimulate POM adaptation? What mechanisms contribute to the hypothesized bi-directional signalling pathway between lactating parent and suckling infant? To what extent does EPOM feeding interfere with this process?
Determining the extent to which weaning reaction research applies to human infant development and lifelong health. How does human milk EGF-mediated GAP inhibition influence immune system development? Is a lack of EGF associated with immune system development disruption? Is a lack of human milk EGF associated with the increased risk of NEC in brief periods of neonatal formula feeding? Characterizing epigenetic signatures of POM feeding at different life stages and identifying critical time windows of POM-mediated epigenetic imprinting. Improving processing technology and storage protocols to maximize the preservation of immunomodulatory POM functions in EPOM and DHM

## Abbreviations

POM: parent’s own milk
Ig: immunoglobulin
HMO: human milk oligosaccharide
NEC: necrotizing enterocolitis
DPOM: direct parent’s own milk
EPOM: expressed parent’s own milk
DHM: donor human milk
TGF: transforming growth factor
IL: interleukin
IGF: insulin-like growth factor
EGF: epithelial growth factor
GCSF: granulocyte colony-stimulating factor
IBD: inflammatory bowel disease
TNF-α: tumor necrosis factor alpha
TLR: toll like receptor
NF-κB: nuclear factor-κB
RORγt+ Treg: RAR-related orphan receptor gamma expressing regulatory T cell
FOS: fructooligosaccharide
GOS: galactooligosaccharides
FA: fatty acid
